# Supplementary material for: Community health worker knowledge, attitudes and practices towards COVID-19: Learnings from an online cross-sectional survey using a digital health platform, UpSCALE, in Mozambique
Source: PLoS One. 2021 Feb 10;16(2):e0244924. doi: 10.1371/journal.pone.0244924 (PMC7875419; doi:10.1371/journal.pone.0244924)
Supplement: S1 Annex — (DOCX) [file pone.0244924.s001.docx]

**S1 Annex**

**KAP APE survey to be deployed on upSCALE platform**

Malaria Consortium, in collaboration with the Ministry of Health, is conducting a survey about the Coronavirus (COVID-19) situation in Mozambique. We would appreciate if you can take 10 minutes and complete this survey for us. Please do not start until you have enough time to complete it in one go, and note that you can stop the survey at any time. All the information we obtain will remain strictly confidential. Persons who participate in this survey are supporting the response against the spread of COVID-19.

By ticking the box, you are agreeing that you have read the above information about the survey, and that you voluntarily agree to take part.

[ ] I agree to participate in this survey.

***Sources of knowledge about COVID-19***

1. Where do you hear or see the messages about COVID-19? (Select all that apply)
2. Newspapers
3. Word-of-mouth (e.g. friends, family)
4. Government website
5. Local television
6. Local radio
7. Through upSCALE
8. Health facility
9. Other (please specify)
10. I have not heard any messages about COVID-19

***General attitudes and practices towards COVID-19***

1. Do you adhere to the prevention measures set out by the Ministry of Health? (E.g. regular handwashing, social distancing)
2. Yes – all of them (*Skip to question 4*)
3. No
4. Some of them or sometimes
5. Don’t know (*Skip to question 4*)
6. What is preventing you from adhering to the prevention measures set out by national health authorities?
7. On a scale of 1 to 5, how able to protect yourself from COVID-19 do you feel? (1 = not at all, 2 = slightly able, 3 = able, 4 = very able, 5 = fully)
   (*If answered 1, 2 or 3,* s*kip to question 6*)

1 2 3 4 5

1. What is preventing you from fully protecting yourself against COVID-19? (E.g. shortage / lack of PPE, insufficient information)

***General knowledge of COVID-19***

1. What are the *three* main clinical symptoms of COVID-19? (Select three)
2. Fever
3. Headache
4. Shortness of breath
5. Dry, persistent cough
6. Conjunctivitis
7. Fatigue
8. Diarrhoea
9. Loss of speech or movement
10. Which of the below are at risk groups for COVID-19? (Select all that apply)
11. Elderly individuals (aged >70)
12. Pregnant women
13. Those with chronic illnesses (e.g. heart disease, diabetes)
14. Children
15. Obese individuals
16. Which of the following are methods of preventing infection with COVID-19? (Select all that apply)
17. Washing hands regularly with soap and water, or cleaning them with alcohol-based hand rub
18. Wearing a facemask
19. Avoid touching your face
20. Covering your mouth and nose when coughing or sneezing with the inside of the elbow or tissue
21. Stay home if you feel unwell
22. Practice physical distancing by avoiding unnecessary travel, staying away from groups exceeding 10 people and keeping 1.5m apart from others
23. How is COVID-19 transmitted? (Select all that apply)
24. Through the air (airborne)
25. Contact with contaminated objects and surfaces
26. Respiratory droplets
27. Direct contact through touching infected persons (e.g. handshake, hug)
28. Emptying latrines and handling of waste
29. What is the *minimum* length of handwashing time recommended to effectively prevent onward transmission of COVID-19?
30. 10 seconds
31. 20 seconds
32. 30 seconds
33. 60 seconds
34. A person infected with COVID-19 who does not show symptoms cannot spread the coronavirus.
35. True
36. False
37. If you have symptoms of COVID-19, what measures should be taken? (Select all that apply)
38. Self-isolate by staying at home for at least 7 days
39. Get plenty of rest
40. Stay hydrated and take paracetamol
41. Contact your local health facility or Alô Vida via telephone
42. Wear a facemask
43. Monitor your symptoms regularly
